# Triticale Improvement: Mining of Genes Related to Yellow Rust Resistance in Triticale Based on Transcriptome Sequencing

**DOI:** 10.3389/fpls.2022.883147

**Published:** 2022-05-09

**Authors:** Fangyuan Zhao, Kuiju Niu, Xinhui Tian, Wenhua Du

**Affiliations:** College of Grassland Science, Key Laboratory of Grassland Ecosystem (Ministry of Education), Pratacultural Engineering Laboratory of Gansu Province, Sino-U.S. Centers for Grazing Land Ecosystem Sustainability, Gansu Agricultural University, Lanzhou, China

**Keywords:** triticale, transcriptome, yellow rust, CYR34, resistance

## Abstract

Yellow (stripe) rust caused by *Puccinia striiformis* f. sp. *tritici* (*Pst*) is a major destructive fungal disease of small grain cereals, leading to large yield losses. The breeding of resistant varieties is an effective, sustainable way to control yellow rust. Elucidation of resistance mechanisms against yellow rust and identification of candidate genes associated with rust resistance are thus crucial. In this study, seedlings of two *Triticosecale* Wittmack cultivars, highly resistant Gannong No. 2 and susceptible Shida No. 1, were inoculated with *Pst* race CYR34. Transcriptome sequencing (RNA-seq) was then used to investigate their transcriptional responses against pathogen infection before and after the appearance of symptoms—10 and 20 days after inoculation, respectively. According to the RNA-seq data, the number of upregulated and downregulated differentially expressed genes (DEGs) in the resistant cultivar was greater than in the susceptible cultivar. A total of 2,560 DEGs commonly expressed in the two cultivars on two sampling dates were subjected to pathway analysis, which revealed that most DEGs were closely associated with defense and metabolic activities. Transcription factor enrichment analysis indicated that the expressions of NAC, WRKY, and FAR1 families were also significantly changed. Further in-depth analysis of resistance genes revealed that almost all serine/threonine-protein kinases were upregulated in the resistant cultivar. Other genes related to disease resistance, such as those encoding disease-resistance- and pathogenesis-related proteins were differentially regulated in the two cultivars. Our findings can serve as a resource for gene discovery and facilitate elucidation of the complex defense mechanisms involved in triticale resistance to *Pst*.

## Introduction

Triticale (×*Triticosecale* Wittmack) is a synthetic hybrid derived from a cross of wheat (*Triticum* sp.) and rye (*Secale* sp.). This hybrid is high yielding and has favorable agronomic characteristics compared with wheat and rye, such as excellent performance in poor and acidic soils or under cold environments (González et al., 2005). Triticale has been developed into a multi-purpose grain-forage species and can be used as a winter cover crop prior to planting of grain crops (Baron et al., 2015). Various diseases and insect pests lead to huge losses in the yield and quality of triticale, among which, yellow rust caused by *Puccinia striiformis* f. sp. *tritici* (*Pst*) has caused serious damage in cool climates (Gyawali et al., 2017). The pathogen prefers humid, cool habitats (3–15°C) and can overwinter on autumn-sown plants (Sodkiewicz et al., 2009). Because chemical and agronomic control methods are not effective when climatic conditions are favorable for the pathogen, the use of resistant cultivars is the most efficient, economically viable, environmentally friendly means of controlling the disease (Ren et al., 2015).

To be pathogenic, an organism must pierce through the cell wall and often the host plasma membrane as well (Zhang et al., 2014). Plants have evolved defense mechanisms to protect themselves from pathogen infection (Jones and Takemoto, 2004). In particular, plants typically use basal and resistance (*R*) gene-mediated defense mechanisms to mount a defensive response to pathogen attack that delays or arrests potential pathogen growth through pathogen-associated molecular pattern-triggered immunity (PTI) and effector-triggered immunity (ETI) systems (Jones and Dangl, 2006). *R* genes are the most effective weapons against pathogen invasion because they can specifically recognize the corresponding pathogen effectors or associated protein (s) that activate plant immune responses at the infection site. Although *R* genes are diverse, most contain conserved motifs, such as nucleotide-blinding site (NBS), leucine-rich repeat (LRR), toll protein and mammalian interleukin-1 receptor (TIR), coiled-coil (CC) or leucine zipper (LZ) structure, transmembrane domain (TM), and protein kinase domain (PK) motifs (Liu et al., 2007).

When plants are infected with various pathogens, the concentrations of endogenous hormones playing crucial roles in regulating plant immune responses, such as salicylic acid (SA), jasmonic acid (JA), and ethylene (ET), are altered (Bari and Jones, 2009). SA is generally involved in the activation of defense against biotrophic pathogens, whereas JA and ET are usually associated with defense against necrotrophic ones (Glazebrook, 2005). Other plant hormones previously implicated in plant growth and development, such as abscisic acid (ABA), auxins (indole-3-acetic acid, IAA), and brassinosteroids (BRs), also play vital roles in the regulation of the immune signaling network and the molding of plant–pathogen interactions, which results in a complex orchestration of plant immunity (Robert-Seilaniantz et al., 2011; Pieterse et al., 2012). Transcription factors (TFs), which control transcriptional regulation—an important method of intracellular regulation—are also involved in plant defense against pathogens. Multiple members of many TF families, such as AP2/ERF, NAC, MYB, and WRKY, participate in pathogen response (Ramamoorthy et al., 2008).

High-throughput sequencing is an efficient and economical molecular analysis strategy. RNA sequencing (RNA-Seq) has been widely applied to investigate plant–pathogen interactions at the transcriptional level in crop and forage plants, including wheat, rice (*Oryza sativa* L.), and alfalfa (*Medicago sativa* L.). This approach has also been used to identify metabolic pathways related to disease resistance, such as phenylpropanoid biosynthesis, plant–pathogen interaction, and plant hormone signal transduction pathways (Yuan et al., 2019). Dobon et al. (2016) performed a RNA-seq time-course analysis of susceptible and resistant wheat hosts infected with *Pst*, which has provided a framework to help clarify how *Pst* causes yellow rust in wheat. Recent studies have examined morphological variation and biochemical and physiological components of triticale. The functions of disease-resistance genes associated with powdery mildew, fusarium head blight, and slow rust have also been reported (Audenaert et al., 2014). The transcriptional responses associated with yellow rust resistance in triticale, a host of *Pst*, have not been reported, and the mechanism of rust resistance in this species is still unclear.

In this study, we used RNA-seq to investigate transcriptional regulation in yellow-rust resistant and susceptible triticale cultivars on two different dates following *Pst* infection. We also analyzed the key defensive metabolic pathways of triticale during *Pst* infection. Our identification of responsive genes in the two triticale cultivars after inoculation has helped elucidate molecular mechanisms underlying the response of triticale to yellow rust caused by *Pst* and has provided valuable genetic information for further functional genomic studies of triticale resistance to yellow rust.

## Materials and Methods

### Plant Materials and Yellow Rust Pathogen Races

Triticale cultivars Gannong No. 2 (henceforth, Gannong 2) and Shida No. 1 (henceforth, Shida 1) were provided by the College of Grassland Science, Gansu Agricultural University, China. The yellow rust *Pst* race CYR34 was supplied by the Institute of Plant Protection, Gansu Academy of Agricultural Science, China. Gannong 2 and Shida 1 infected by *Pst* race CYR34, representing incompatible and compatible reactions, respectively.

### Plant Cultivation, Inoculation Conditions, and Infection Assessment

Triticale plants were cultivated in pots (14 cm × 14 cm × 12 cm; 10 plants/pot) filled with 1:1 (v/v) field soil and vermiculite, which was supplemented before sowing with diammonium phosphate (0.45 g per pot). Plump, healthy triticale seeds of consistent maturity were sown in the filled pots, which were irrigated at 2-day intervals. Plants were cultured at 27°C/24°C (day/night) under a 16/8-h photoperiod (light/dark) and a relative humidity of 65%. Inoculation with *Pst* race CYR34, the most widespread race in China, was carried out 15 days later using the standard inoculation procedure detailed in Bozkurt et al. (2007). Briefly, plants during inoculation were incubated at 10°C under extremely high humidity for 24 h in the dark and then transferred to normal growth conditions (16-h light at 15°C–17°C and 8-h dark at 14°C–16°C). Fifteen days after inoculation, plant infection types (ITs) were assessed on a scale of 0–4 (Roelfs et al., 1992), where 0, 0; 1, 2, 3, and 4 indicate immunity, near immunity, moderate resistance, high resistance, moderate susceptibility, and high susceptibility to yellow rust, respectively (Huang et al., 2014). Leaves of Gannong 2 and Shida 1 seedlings inoculated with CYR34 with different phenotypes were collected both prior to before and after the sporulation (susceptible cultivar Shida 1), namely, 10 and 20 days after inoculation, respectively. Samples collected from Gannong 2 on days 10 and 20 were designated as R10 and R20, respectively, with the corresponding samples from Shida 1 labeled as S10 and S20. All fresh samples were immediately frozen in liquid nitrogen and stored at −80°C until RNA extraction.

### RNA Extraction, cDNA Library Construction, and Illumina Sequencing

Total RNA was extracted from leaf samples using an RNA simple Total RNA kit (Tiangen, Beijing, China). The extracted RNA was quantified on a NanoDrop ND-1000 instrument (Thermo Scientific), and RNA integrity was determined by 1% agarose gel electrophoresis. Construction of RNA-Seq and cDNA libraries was carried out with 1 μg RNA per sample using a NEBNext UltraTM RNA Library Prep Kit for Illumina (NEB, United States). Sequencing of the eight generated libraries (R10, S10, R20, and S20, with two biological replicates per sample) was performed on an Illumina HiSeq 2000 system (Illumina, San Diego, CA, United States) by Biomarker Technologies Co. (Beijing, China).

### Sequence Read Mapping, Transcript Assembly, and Functional Annotation

Clean reads were obtained by removing adapter and poly-N-containing reads and low-quality sequences from the raw data. Q30 values, GC contents, and sequence duplication levels of the filtered data were then calculated. The clean reads were subsequently mapped to the wheat reference genome.^1^ Reads with no more than one mismatch were further analyzed and annotated against the reference genome using Hisat2 (Kim et al., 2015). The mapped reads were assembled with StringTie software (Pertea et al., 2015) and used for mining new transcripts and genes of triticale. The assembled genes were aligned against several protein databases, including Nr, Swiss-Prot, Kyoto Encyclopedia of Genes and Genomes (KEGG), COG/KOG, Gene Ontology (GO), Pfam and eggNOG, using BLASTx (*E*-value <1 × 10^−5^; Altschul et al., 1997).

### Differential Expression and Gene Ontology/Kyoto Encyclopedia of Genes and Genomes Enrichment Analyses

Differentially expressed genes (DEGs) were determined according to their expression abundances in different treatment groups, with new transcript and gene expressions calculated by the expected number of fragments per kilobase of transcript sequence per million base pairs sequenced (FPKM) method (Xue et al., 2010). GO enrichment analysis of DEGs was carried out using the GOseq R package based on Wallenius’ noncentral hypergeometric distribution (Young et al., 2010). KEGG pathway enrichment analysis was performed using KOBAS software with a corrected *p* < 0.05 (Mao et al., 2005). A comparative heatmap analysis of DEGs in two triticale cultivars (Gannong 2 and Shida 1) at 10 vs. 20 days after *Pst* infection was also carried out using the Biomarker Cloud platform.^2^

### qRT-PCR Validation of DEGs

To experimentally evaluate the RNA-Seq results, we conducted a qRT-PCR analysis. Total RNA was extracted from leaf samples and reverse transcribed into cDNA using a PrimeScript RT reagent Kit with gDNA Eraser (Takara, Japan). qRT-PCR amplifications were conducted using SYBR Green Real-Time PCR Master Mix (Takara) on a LightCycler96 thermal cycler (Roche, Switzerland). Sixteen DEGs were randomly selected from kinase-related genes, disease-resistance protein genes and transcription factor that were upregulated in the resistant cultivar both at 10 and 20 days after inoculation, which were amplified with specific primers designed using Primer 5.0 software (Table 1), with three biological replicates performed per sample, and each with three technical repeats. The β-actin gene was used as an internal standard. Relative transcript levels of the selected genes were calculated by the 2^-ΔΔCt^ method (Livak and Schmittgen, 2001).

**Table 1:** Primers used for validation of RNA-seq values by qRT-PCR.

Gene ID	Forward primer	Reverse primer
TraesCS2D02G462000	CTCGCTGACCGGAGACATAC	TTCGTCAGGTTGCCGATCTC
TraesCS2A02G574800	CCACCTTTGGGAGGCTCAAT	GTTGCTGGCAAGGTTCAGTG
TraesCS5A02G487000	TGCTTGGATGGTCACGTCTC	TGCGTTCTCCTTGACAGCAT
TraesCS5A02G445700	GCATATGCAAGAACAGGCCG	TACCAACAATCGCCTTGGCT
TraesCS5B02G063600	CCCCTGCGATTATGCGTTTG	GGCAGAGACTGTTGGAGCTT
TraesCS7A02G425900	ACTACCAGACTGGGCAGCTA	CTTCAAGAACTTGTGCCGCC
TraesCS7D02G131800	AGTTGGAGATCTTGGGCGTG	AGACCATCAGTGAGTGCAGC
TraesCS7A02G069600	CCAATCTGACACCCAGTCCC	GTTGCTGCTCATTGGTGCAA
TraesCS4A02G482900	GTGTGGGCTAGCAGAATGGT	TCTGGCACTTCTTGACGACC
TraesCS1B02G284600	TCCCTTGCCTACCTGCTACT	CGATGCAGTAGGTCGATGCT
TraesCS4B02G387600	CGAAGGTTGGTGTGGCAAAG	TTGGTCACTGTTGTGCAGGT
TraesCS7D02G031000	TCCCTGCGCTCTCTTAGTCT	CCACTCGGGCATCTTTCCTT
TraesCS4B02G329100	GACGCTCGTCTTCTACCAGG	CCATCGCAAATGCTCTCTGC
TraesCS7B02G472600	TACCTCAAGCACCACGTCAC	GCACGAGGAAGTACCACTCC
TraesCS5A02G225600	CCCAGAGCCTACTTCCGATG	GATGGAGATGGAGCATGGCA
TraesCS5A02G259000	TTCCTGGAGTCTGCCACAAG	CCTGATGGTGTGCAGCGTA
β-Actin	GGAAAAGTGCAGAGAGACACG	TACAGTGTCTGGATCGGTGGT

## Results

### Evaluation of Resistance

**Figure 1:**
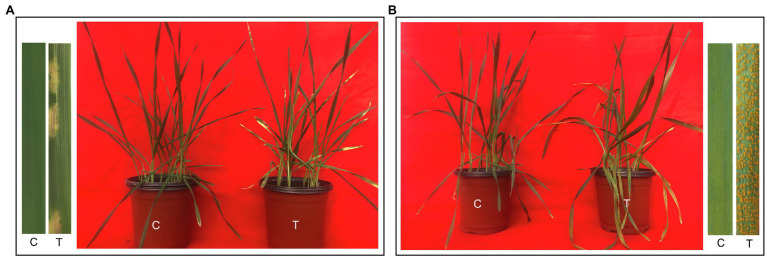
Phenotypes of triticale cultivars Gannong 2 and Shida 1 at 15 days after inoculation with *Pst* race CYR34. **(A,B)** Responses of untreated and treated seedlings of Gannong 2 **(A)** and Shida 1 **(B)**. C, untreated control; T, treatment.

Fifteen days after inoculation, Gannong 2 and Shida 1 were photographed and assessed for disease severity (Figure 1). Compared with non-inoculated control seedlings, Gannong 2 seedlings infected with CYR34 exhibited a few necrotic stripes, but no sporulation, and were recorded as highly resistant, with an IT score of 1. In contrast, Shida 1 developed heavy sporulation after inoculation; it was highly susceptible to yellow rust race CYR34, with an IT score of 4. It was consistent with the identification results of these two cultivars at seedling stage by the Institute of Plant Protection, Gansu Academy of Agricultural Science, China. In addition, Gannong 2 was a resistant cultivar and Shida 1 was a susceptible cultivar at the adult plant stage, according to the identification by the institute.

### Transcriptome Sequencing Analysis and Functional Annotation

**Figure 2:**
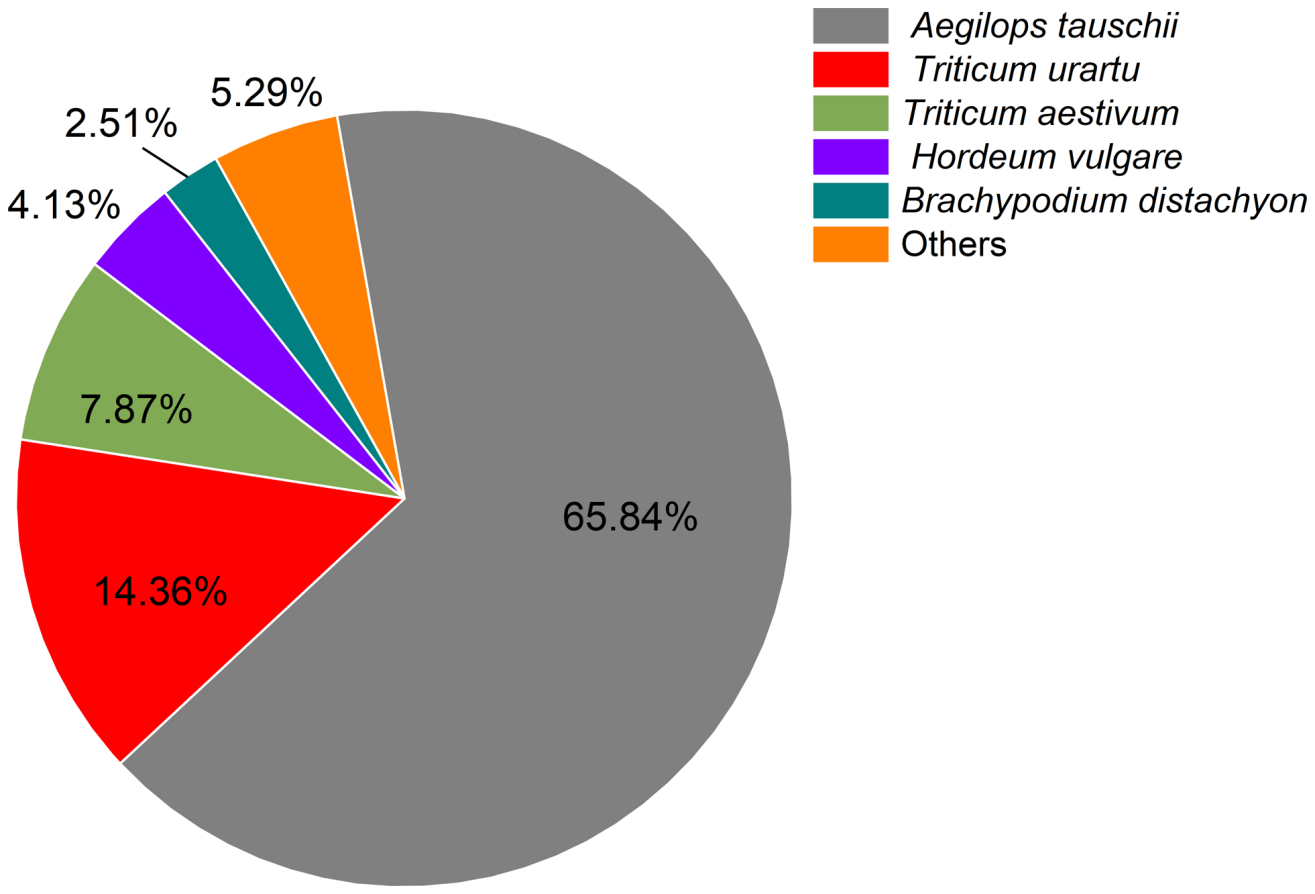
Distribution of matching homologous species based on Nr annotation.

Filtering of raw reads to remove adaptor-containing and low-quality reads yielded 59.30 Gb clean reads for subsequent analyses. An average of 5.75 Gb clean reads were generated per sample, Q30 and GC values ranged from 94.27%–95.77% and 53.41%–56.96%, respectively, and *r*^2^ values between replicates varied between 0.974 and 0.990, these numbers, which reflect both a high correlation and slight variability among biological replicates, demonstrate the reliability of the RNA-seq data. On average, 65.35% of total reads were mapped to the wheat reference genome. A total of 33.20% (38,849), 72.44% (84,761), 30.28% (35,438), 45.80% (53,593), 75.22% (88,018), 66.63% (77,969), 88.11% (103,104), and 99.81% (116,796) of transcripts were successfully annotated against COG, GO, KEGG, KOG, Pfam, Swiss-Prot, and Nr databases, respectively, indicating that most transcripts had significant matches to genes of known function. The most abundant transcripts (79,090) were those longer than 1,000 bp (Table 2). Further investigation of BLAST results against the Nr database revealed that triticale transcripts were significantly similar to *Aegilops tauschii* proteins (65.85%), followed by *Triticum urartu* (14.36%) and *Triticum aestivum* (7.87%). In addition, other transcripts were similar to those of proteins of gramineous species, including *Hordeum vulgare* (4.13%) and *Brachypodium distachyon* (2.51%; Figure 2).

**Table 2:** Summary of the gene functional annotation and the length distribution of the transcripts.

Database	Number of annotated genes	The length distribution of the transcripts		
		<300	300–1,000	≥1,000
COG	38,849 (33.20%)	431	5,504	32,914
GO	84,761 (72.44%)	2,733	20,501	61,527
KEGG	35,438 (30.28%)	1,037	7,806	26,595
KOG	53,593 (45.80%)	863	10,320	42,410
Pfam	88,018 (75.22%)	1,687	19,334	66,997
Swiss-Prot	77,969 (66.63%)	1867	16,702	59,400
eggNOG	103,104 (88.11%)	2,897	25,499	74,708
Nr	116,796 (99.81%)	5,039	32,724	79,033
All	117,016	5,057	32,869	79,090

### Identification of DEGs

**Figure 3:**
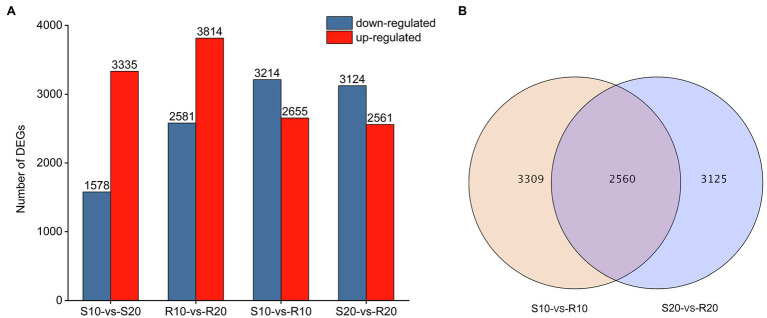
Genes differentially expressed in two triticale cultivars at different treatment time points. **(A)** Number of differentially expressed genes (DEGs) per cultivar at each time point. **(B)** Venn diagram depicting the number of DEGs and their overlap at each time point.

DEGs were identified by comparing expression levels in plants of susceptible Shida 1 and resistant Gannong 2 at 10 and 20 days after inoculation. In each comparison, a FDR threshold of 0.05 and a log_2_ (fold change) ≥ 1 were used as criteria for determination of DEGs. The transcript abundance of each gene was further calculated in terms of FPKM and analyzed with Cufflinks (Trapnell et al., 2010). After inoculation with CYR34, 3,335 and 1,578 genes were, respectively, upregulated and downregulated in S10-*vs*-S20, whereas 3,814 were upregulated and 2,581 were downregulated in R10-*vs*-R20 (Figure 3A). It shows that the number of upregulated and downregulated DEGs in the resistant cultivar was greater than that of the susceptible cultivar. Compared to susceptible cultivar, a total of 5,869 DEGs (2,655 upregulated and 3,214 downregulated) were identified in the resistant cultivar at 10 days after inoculation (S10-*vs*-R10), and 5,685 DEGs (2,561 upregulated and 3,124 downregulated) were uncovered at 20 days after inoculation (S20-*vs*-R20; Figure 3A). It shows that there was no significant difference between these DEGs that upregulated and downregulated at 10 days and 20 days, respectively. According to a Venn diagram analysis, 2,560 DEGs were common to both S10-*vs*-R10 and S20-*vs*-R20 (Figure 3B).

### GO and KEGG Enrichment Analysis of DEGs After Inoculation With CYR34

The above 2,560 DEGs were subjected to GO and KEGG pathway enrichment analyses. The GO enrichment analysis divided the DEGs into three main categories and 47 sub-categories, namely, 19, 15, and 13 sub-categories related to biological processes, cellular components, and molecular functions, respectively. Most enriched DEGs were classified into the biological process category and were mainly involved in functions associated with metabolic process (GO: 0008152), cellular processes (GO: 0009987), single-organism process (GO: 0044699), biological regulation (GO: 0065007), cell (GO: 0005623), binding (GO: 0005488), and catalytic activity (GO: 0003824; Figure 4). These results suggest that the majority of DEGs are related to multiple metabolic processes.

**Figure 4:**
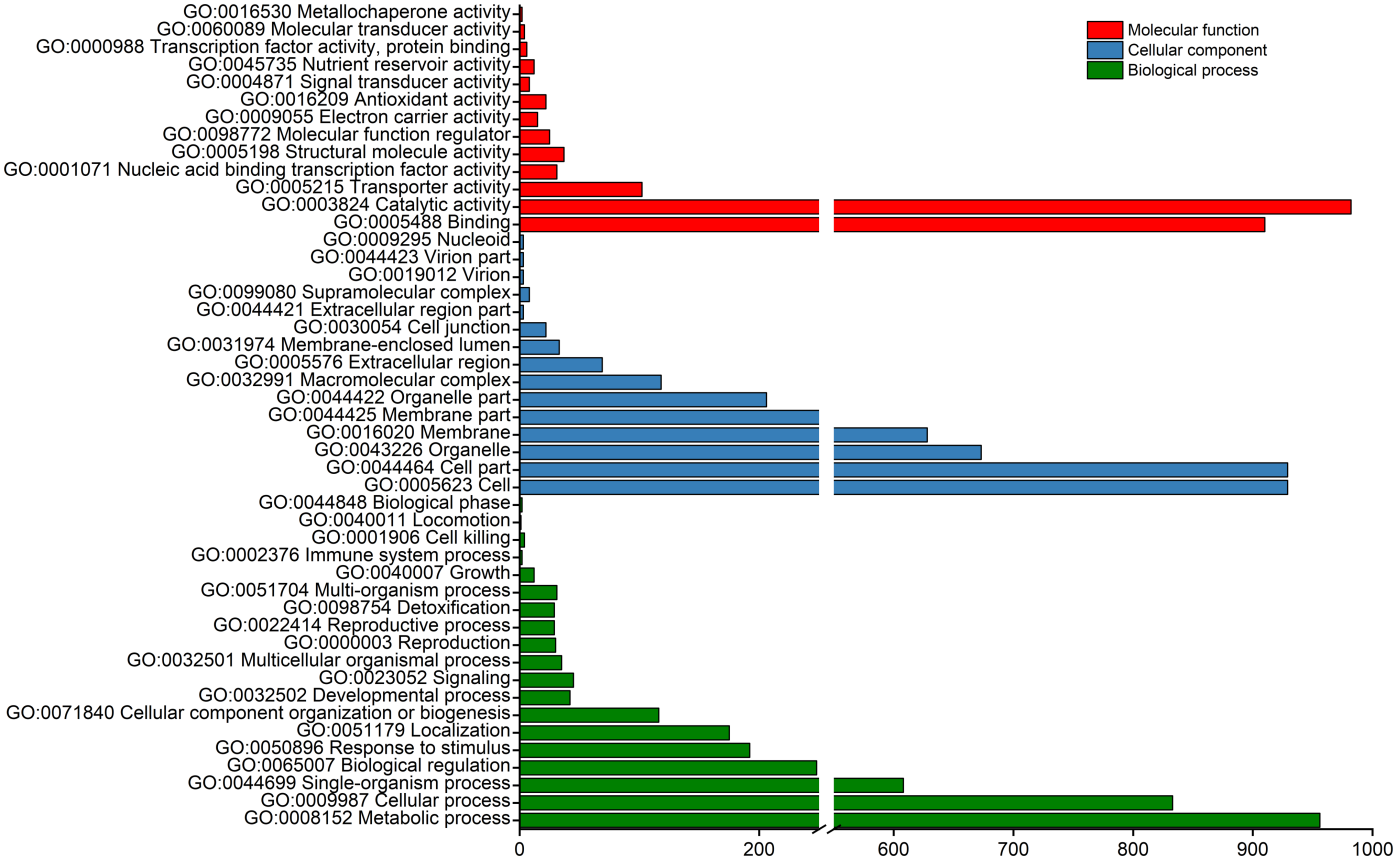
GO enrichment analysis of DEGs. The main GO terms assigned to “biological process,” “cellular component,” and “molecular function” categories are shown.

The KEGG enrichment analysis revealed that several KEGG pathways were significantly enriched after *Pst* infection, including phenylalanine metabolism (ko00360), plant–pathogen interaction (ko04626), plant hormone signal transduction (ko04075), phenylpropanoid biosynthesis (ko00940), biosynthesis of amino acids (ko01230), and carbon metabolism (ko01200) and so on. A total of 898 DEGs were associated with 117 KEGG pathways. The top 20 KEGG pathways based on the number of enriched DEGs are shown in Table 3. Taken together, these results suggest that a range of molecular defense strategies have evolved in triticale. As indicated by these results, the process is complex and likely regulated by the interaction of multiple pathways, such as various hormone signaling and plant–pathogen interaction pathways. We therefore investigated the DEGs involved in these two pathways.

**Table 3:** Kyoto Encyclopedia of Genes and Genomes (KEGG) pathways significantly enriched in DEGs.

KEGG pathway	Number of genes	*p*	ID
Phenylpropanoid biosynthesis	49	0.0003	ko00940
Biosynthesis of amino acids	39	7.41E-05	ko01230
Carbon metabolism	34	0.0101	ko01200
Amino sugar and nucleotide sugar metabolism	29	3.59E-05	ko00520
Starch and sucrose metabolism	25	0.2021	ko00500
Protein processing in endoplasmic reticulum	23	0.4620	ko04141
Ribosome	23	0.9793	ko03010
Phenylalanine metabolism	21	1.01E-08	ko00360
Plant-pathogen interaction	20	0.4177	ko04626
Alpha-linolenic acid metabolism	18	2.56E-05	ko00592
Cysteine and methionine metabolism	18	0.0054	ko00270
Glutathione metabolism	18	0.0677	ko00480
Glyoxylate and dicarboxylate metabolism	17	0.0003	ko00630
Pyrimidine metabolism	17	0.1279	ko00240
Glycine, serine and threonine metabolism	16	0.0007	ko00260
Galactose metabolism	16	0.0100	ko00052
Glycolysis/gluconeogenesis	16	0.1512	ko00010
RNA transport	16	0.4133	ko03013
Plant hormone signal transduction	16	0.9727	ko04075
Pyruvate metabolism	15	0.0075	ko00620

### DEGs Related to Phytohormone Signaling

**Figure 5:**
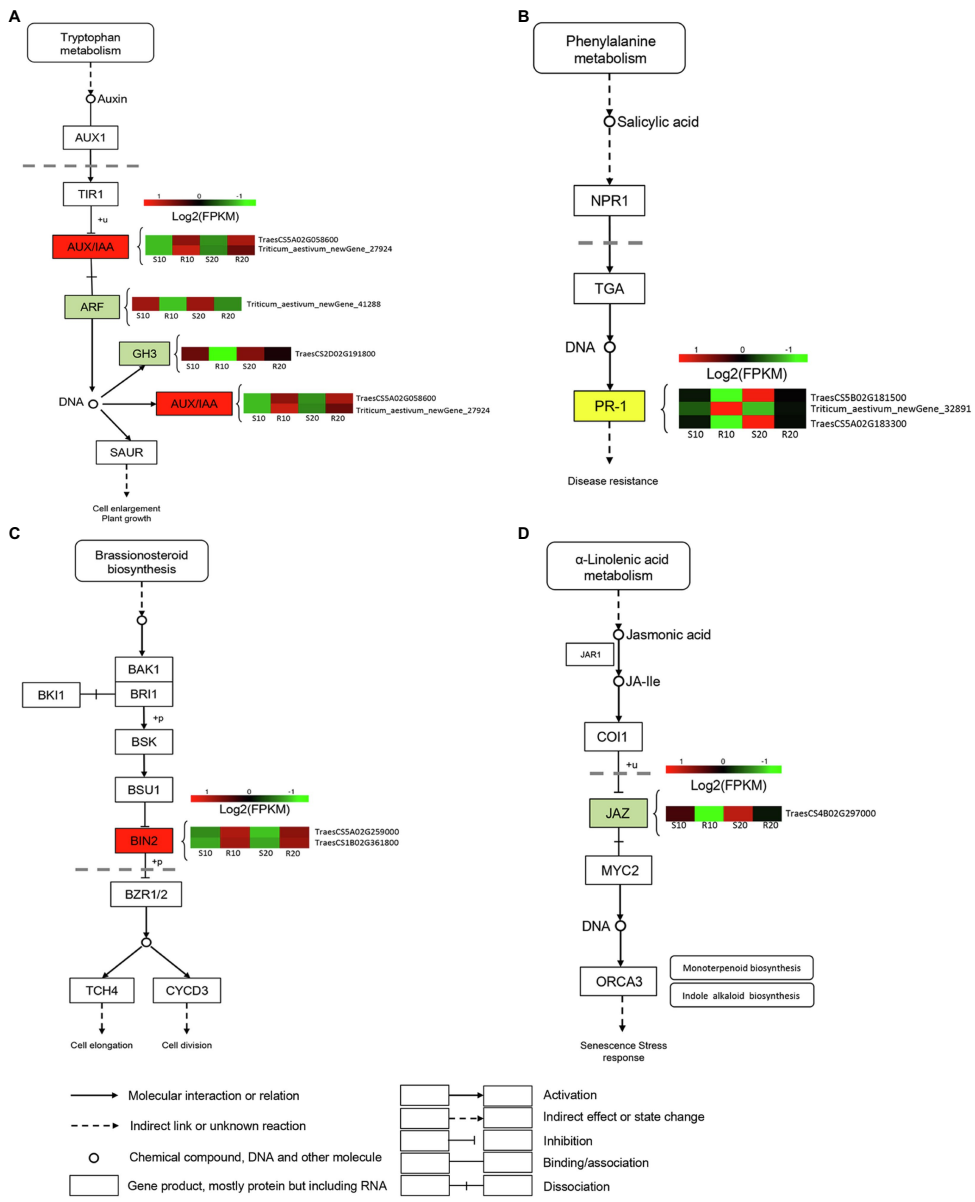
DEGs related to phytohormone signaling transduction pathway. **(A)** Auxin (AUX); **(B)** salicylic acid (SA); **(C)** brassinosteroid (BR); and **(D)** jasmonic acid (JA). Expression values are presented as log_2_ FPKM values (red for upregulated and green for downregulated), and the vertical columns, from left to right, represent S10, R10, S20, and R20. The color of the background indicates the pattern of gene expression: red for upregulation, green for downregulation, and yellow for both upregulation and downregulation.

To identify DEGs associated with hormonal response in triticale leaves infected with *Pst*, we analyzed the expressions of genes involved in hormone signal transduction pathways (Figure 5). Several genes designated as AUX-responsive, including auxin/indole-3-acetic acid (AUX/IAA), auxin-responsive Gretchen Hagen3 (GH3), and auxin response factor (ARF), were differentially expressed between resistant and susceptible cultivars (Figure 5A). Several DEGs involved in SA biosynthesis and signaling were also observed. Three DEGs were annotated as pathogenesis-related protein (PR-1) genes; one was upregulated in the resistant cultivar compared with the susceptible cultivar, and the other two were downregulated (Figure 5B). Two brassinosteroid insensitive 2 (BIN2) genes, involved in BR signaling, were significantly upregulated in the resistant cultivar relative to the susceptible one (Figure 5C). The only identified DEG encoding JAZ protein was downregulated in the resistant cultivar compared with the susceptible one (Figure 5D).

### DEGs Related to Plant–Fungus Interaction

**Figure 6:**
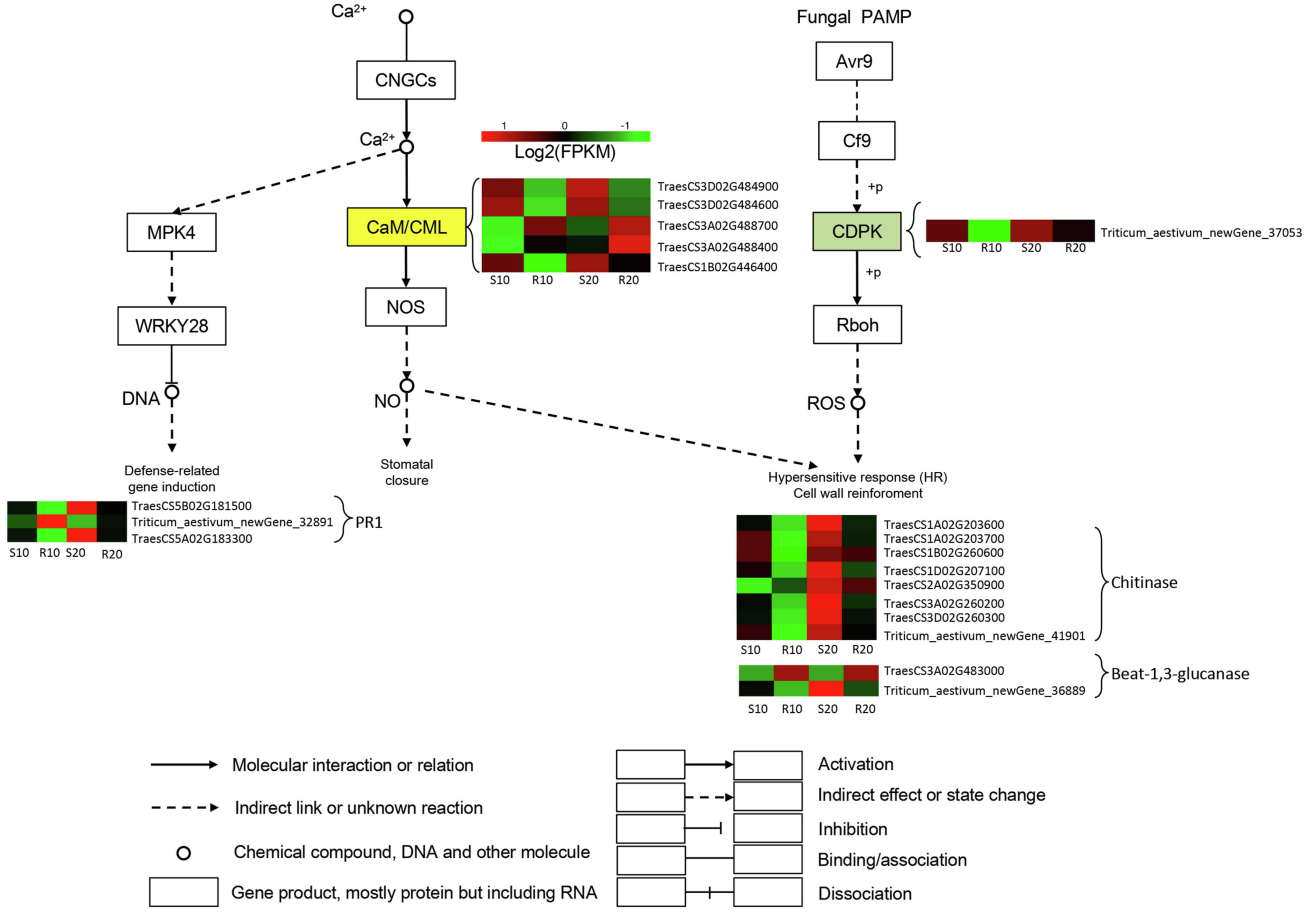
DEGs related to triticale–*Pst* interaction pathway. Expression values are presented as log_2_ FPKM values (red for upregulated and green for downregulated), and the vertical columns, from left to right, represent S10, R10, S20, and R20. The color of the background indicates the pattern of gene expression: red for upregulation, green for downregulation, and yellow for both upregulation and downregulation.

One DEG encoding a calcium-dependent protein kinase (CDPK) involved in induction of hypersensitive response and cell wall reinforcement was significantly upregulated in the susceptible cultivar compared with the resistant one. Furthermore, numerous DEGs encoding chitinases (CHI) and glucanase-related genes were upregulated in the susceptible cultivar relative to the resistant one, and the expressions of these genes gradually increased in both cultivars during *Pst* infection. Genes encoding Crassulacean acid metabolism/calmodulin-like proteins (CaM/CMLs) were differentially expressed between the two cultivars, with two DEGs upregulated in the resistant cultivar, and three upregulated in the susceptible cultivar. The expressions of these five genes gradually increased in both cultivars during *Pst* infection. Defense-related genes were also induced; in particular, one and two PR1 genes were upregulated in resistant and susceptible cultivars, respectively (Figure 6).

### TFs Differentially Expressed in Response to *Pst*

**Figure 7:**
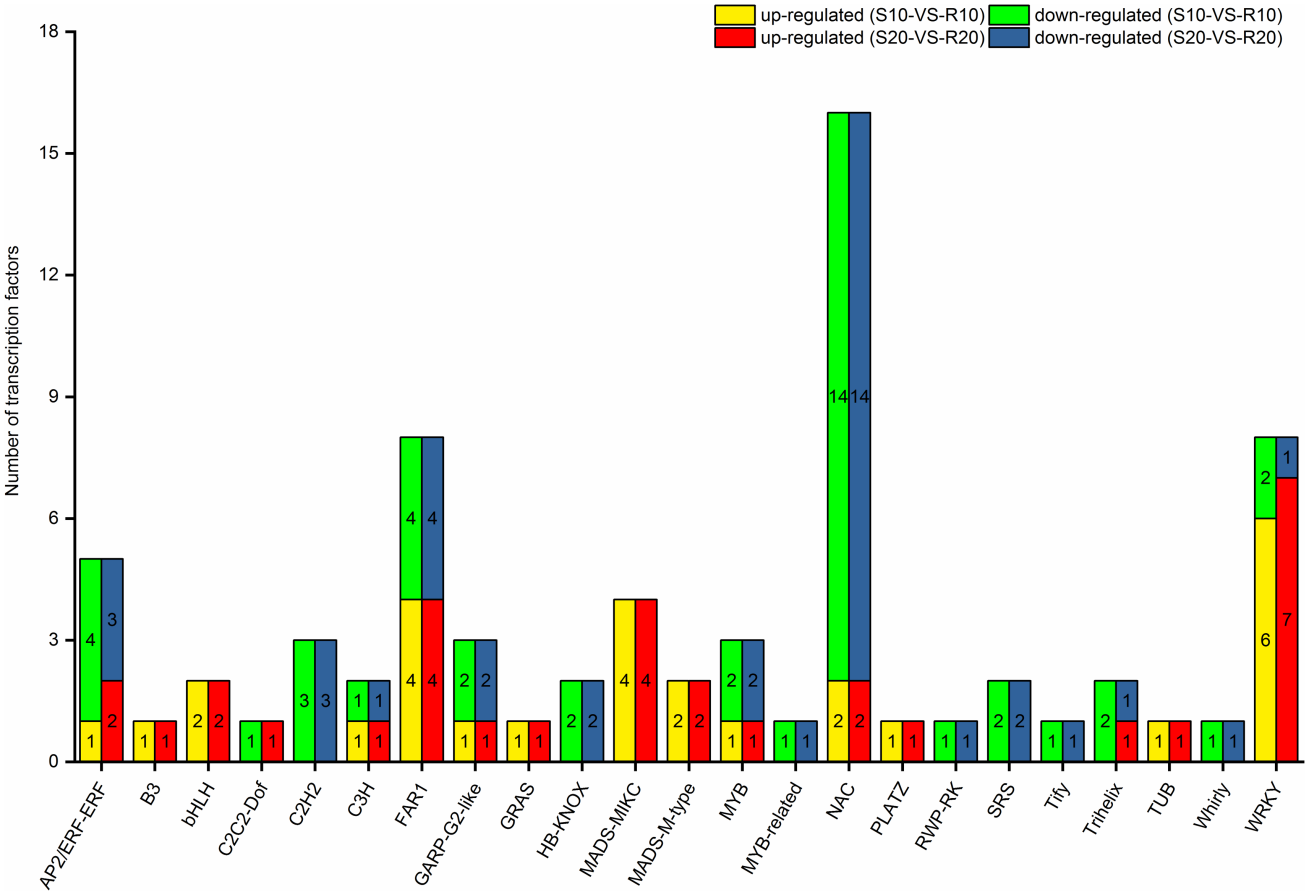
Distribution of differentially expressed transcription factors in triticale leaves infected with *Pst*.

Given that TFs can regulate gene expression by binding to target gene promoters (Singh et al., 2002), an analysis of TFs might provide a basis for understanding the regulatory network operating in triticale seedlings in response to *Pst*. Seventy one of the 2,560 DEGs under *Pst* infection in this study were identified as TF genes. Among them, 28 were upregulated in the resistant cultivar both at 10 and 20 days after inoculation, whereas 39 were downregulated. All the differentially expressed TF genes encoded members of 23 TF families (Figure 7), mainly NAC (16), WRKY (8), FAR1 (8), AP2/ERF-ERF (5), and MADS-MIKC (4) families.

### Characteristics of DEGs Associated With *Pst* Resistance

**Figure 8:**
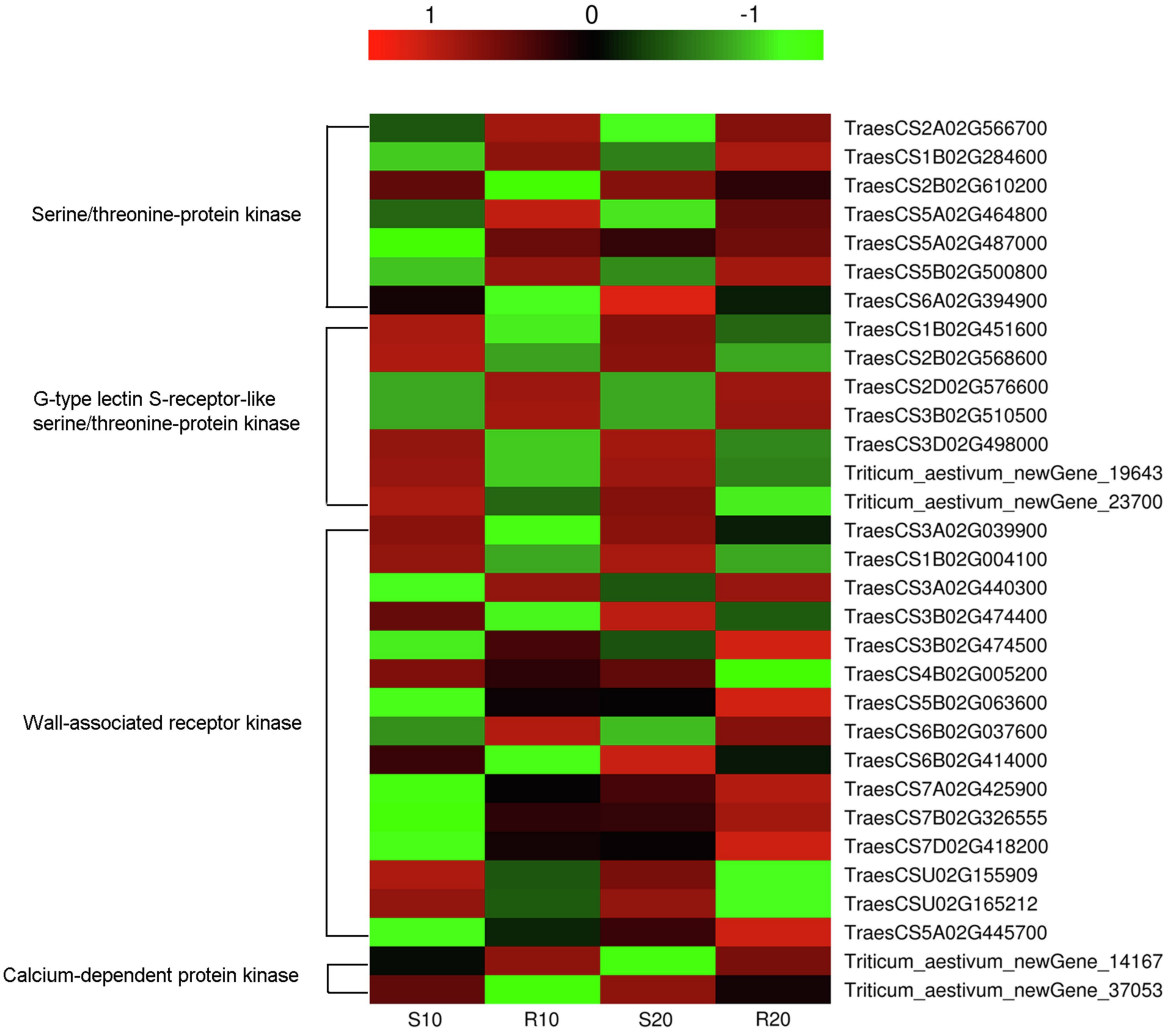
Heatmap of transcript levels of disease-resistance kinases related to *Pst* resistance in leaves of resistant and susceptible triticale cultivars 10 and 20 days after *Pst* infection.

Numerous DEGs, the *R* gene such as those encoding disease-resistance proteins (RGAs, RPP13, and RPM1), and other pathogenesis-related proteins (PR1.1 and PR1-like) may involved in disease-resistance processes, were identified. Moreover, 31 DEGs encoding disease-resistance kinases were also identified, these included genes for serine/threonine-protein kinase (7), wall-associated receptor kinase (15), G-type lectin S-receptor-like serine/threonine-protein kinase (7), and calcium-dependent protein kinase (2). The expression levels of most kinase genes exhibited no significant differences at 10 vs. 20 days after inoculation. The exceptions were TraesCS5B02G063600, TraesCS7A02G425900, TraesCS7B02G326555, TraesCS7D02G418200, and TraesCS5A02G445700, which were weakly expressed before sporulation (10 days after inoculation) but significantly upregulated afterwards in both cultivars (Figure 8). Compared with their expressions in the susceptible cultivar, some of the disease-resistance kinase genes were upregulated (16) in the resistant cultivar, while others were downregulated (15), the genes upregulated and downregulated in the two cultivars were almost the same at 10 and 20 days after inoculation (Figure 8).

### qRT-PCR Analysis of DEGs Associated With *Pst* Resistance

**Figure 9:**
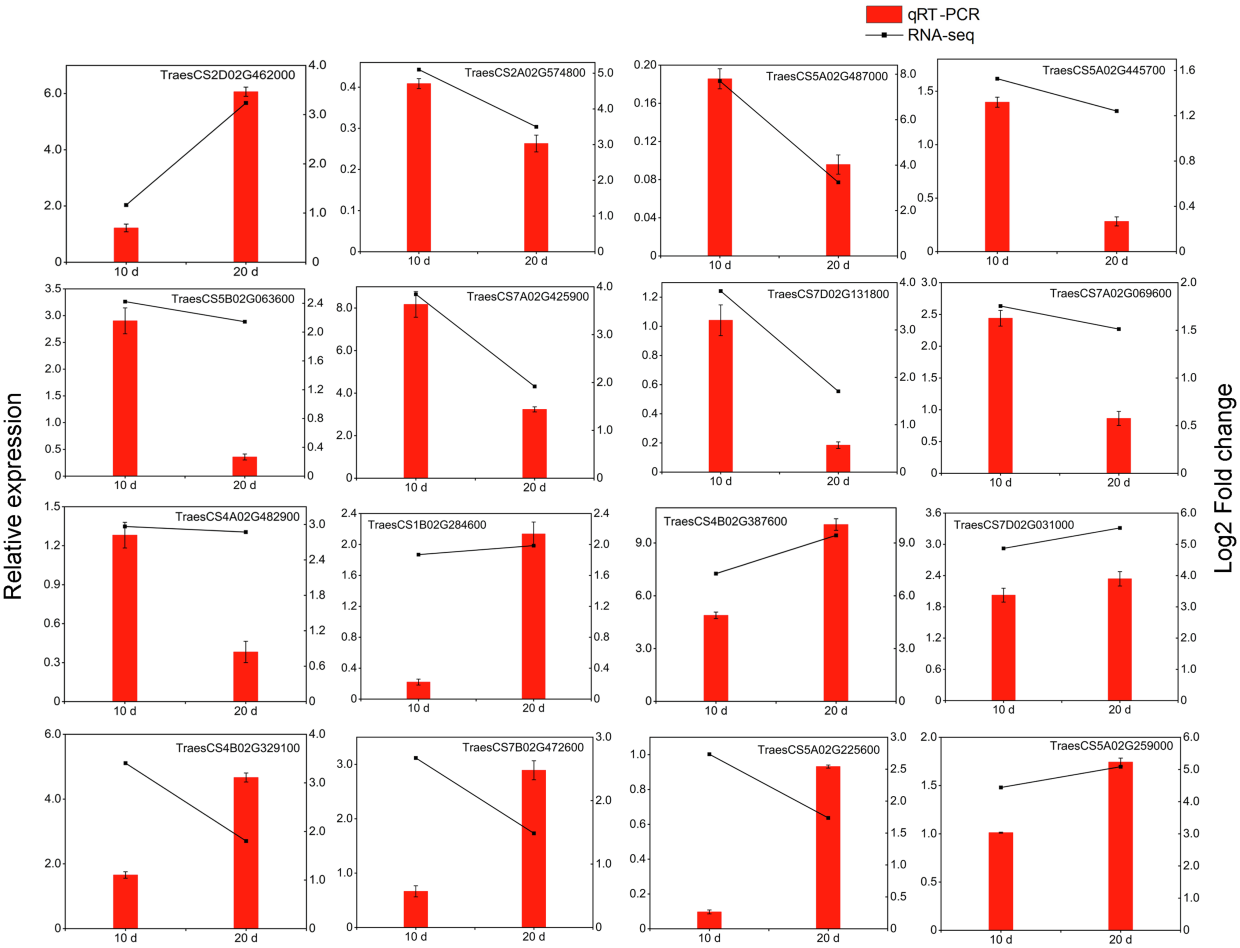
qRT-PCR analysis of selected DEGs in triticale under the yellow rust infection. Red columns indicate relative expression levels determined by qRT-PCR (left *y*-axis), and dashed lines represent transcript level changes (log_2_ fold change) based on FPKM values determined by RNA-Seq (right *y*-axis).

In this study, 16 DEGs (including 10 kinase-related genes, three disease-resistance protein genes and three TFs) were randomly selected for qRT-PCR analysis and were confirmed to be upregulated in the resistant cultivar both at 10 and 20 days after inoculation (Figure 9). Although some differences in the range of expression changes were observed, the expression trends of these genes based on fold changes in relative gene expression as measured by qRT-PCR were basically consistent with their corresponding transcript abundance changes detected by RNA-Seq; this indicates that the expressions of the 16 genes were reliable in the present study (Figure 9). All of these genes were more highly expressed in the resistant cultivar than in the susceptible cultivar under *Pst* infection. The four most significantly expressed genes (TraesCS2D02G462000, TraesCS7A02G425900, TraesCS4B02G387600, and TraesCS7D02G0 31,000, corresponding to *FLS2*, *WAK5*, *LecRK-IX.2*, and *RPM1*, respectively) may play an active role during PTI or ETI.

## Discussion

Yellow (stripe) rust is one of the most destructive diseases of plants, causing severe losses in small grain cereals, including triticale (Losert et al., 2017). The highly aggressive race CYR34 currently dominates the yellow rust population in wheat and triticale in China (Huang et al., 2019), therefore, clarifying the resistance level of triticale to CYR34 and screening the yellow rust resistance-related genes is of great significance in triticale yellow rust-resistance breeding. RNA-seq has provided a new avenue to study the responses of plants to pathogen. Until the present study, however, RNA-seq had not been exploited to investigate yellow rust resistance genes in triticale, and the mechanism of rust resistance in triticale was still unknown. We therefore used RNA-seq to analyze the expression of *Pst*-related genes in resistant and susceptible triticale cultivars at later stages (before and after sporulation of susceptible cultivar) to identify changes in gene expression that can be linked to key aspects of the infection process.

After inoculation with *Pst*, symptoms of chlorosis are generally observed 6–8 days later, whereas sporulation commences approximately 12–14 days post-inoculation under favorable conditions (Chen et al., 2014). In the present study, Shida 1 began to sporulate 13–15 days after inoculation, and the area of the spores on the leaves gradually increased, compared to Shida 1, Gannong 2 had only a few necrotic stripes, but no sporulation. Therefore, Shida 1 was identified as a highly susceptible cultivar, and Gannong 2 was a resistant cultivar, this is consistent with the symptoms described by Huang et al. (2014). Samples collected 10 and 20 days after inoculation were therefore used for an analysis of triticale defense mechanisms during infection. In the present study, DEGs in resistant and susceptible triticale were analyzed by RNA-Seq after inoculation with *Pst*. As a result, we identified 2,560 genes that were differentially expressed in the two cultivars during *Pst* infection. In a previous study, Yuan et al. (2019) found that more DEGs were induced in a *Fusarium graminearum*-resistant line than in a susceptible one. In our study, we found no significant difference in the number of DEGs before and after sporulation, but the number of DEGs in the resistant cultivar (Gannong 2) was greater than that of the susceptible cultivar (Shida 1). We thus hypothesize that a stronger defense response was activated in the resistant cultivar than in the susceptible one, similar to the results of Yuan et al. (2019). Our findings are also consistent with the results of Dobon et al. (2016), who showed that the *Pst* pathogen suppresses defense-related gene expression in a compatible interaction to enable successful colonization.

KEGG pathways enriched in DEGs were also analyzed. In a previous study, Hao et al. (2016) found that “phenylpropanoid biosynthesis” pathway were involved in the yellow rust defense response of wheat in the early stage. In our study, the most significant enrichment occurred in the “phenylpropanoid biosynthesis” pathway, and the number of DEGs was largest in this pathway, which indicated that this pathway was also involved in the response of triticale to *Pst* infection in a later stage. In addition, the number of DEGs associated with “plant–pathogen interaction” and “plant hormone signal transduction” was also higher in our study. Plant hormones such as SA, JA, and ET play major roles in the regulation of plant defense responses (Camehl et al., 2013). Auxin and other plant hormones have also been reported to be involved in the regulation of plant resistance to pathogens (Spoel and Dong, 2008). Dobon et al. (2016) have identified response pathways related to the wheat stress hormones SA, JA, ethylene, and ABA, whose balance is fine-tuned to regulate plant innate immunity. In the present study, we therefore focused on plant hormone signal transduction pathways (Ko04075). Annotation of DEGs classified 16 genes into plant hormone signal transduction pathways, including those associated with auxin, BR, JA, and SA. AUX/IAA proteins are positive regulators of disease resistance, but auxin signaling is inactivated by AUX/IAA protein binding (Yang et al., 2014). After infection of the resistant cultivar (Gannong 2) by *Pst*, AUX/IAA gene expressions were upregulated, which may have suppressed the auxin signaling pathway to provide resistance to *Pst*. BRs promote plant growth and development and have a complex positive role in the plant immune defense response of Selaginella and Arabidopsis (Cheon et al., 2013), but they play a negative role in the interaction between rice and pathogens (He et al., 2017). De Vleesschauwer et al. (2012) have reported that BRs boost the root immunity of rice against infection by the pathogen *Pythium graminicola*. Brassinosteroid-INsensitive 2 (BIN2) is an Arabidopsis GSK3-like kinase that negatively regulates the intracellular signal transduction of BRs (Peng et al., 2010). In the present study, we found that the two DEGs encoding BIN2 were strongly upregulated in the resistant cultivar, possibly inhibiting BR signaling through over-expression of the *BIN2* gene. The role of BR in the interaction between triticale and *Pst* needs to be studied further. JAZ proteins act as negative regulators of JA signaling by suppressing MYC2 proteins (Yang et al., 2014), but JA is a positive regulator of immunity against necrotrophic pathogens (Tsuda and Somssich, 2015). In our study, we found that one *JAZ* gene was downregulated in the resistant cultivar (Gannong 2) but upregulated in the susceptible cultivar (Shida 1). We hypothesize that this downregulation is due to active suppression of *JAZ* gene expression by *Pst* in the resistant cultivar. NPR1 is a key regulator of systemic acquired resistance (SAR)-related PR gene expression, and NPR1-overexpressing plants have enhanced resistance to various pathogens without constitutively expressing the PR genes (Kinkema et al., 2000). No differential expression of NPR1 associated with SA signaling was detected in our study, and downstream PR-1 genes in resistant cultivar (Gannong 2) were both upregulated and downregulated. This result is in keeping with observations showing that defense responses to necrotrophic organisms are SA-independent (Kinkema et al., 2000).

A plant–pathogen interaction pathway (Ko04626) is also a component of the plant environmental adaptation and immune system (Yuan et al., 2019). In the present study, 20 DEGs were assigned to this plant–pathogen interaction pathway. Among them, one DEG encoding CDPK was identified and found to be significantly upregulated in the susceptible cultivar (Shida 1). We hypothesize that susceptible varieties can still perceive the fungus at later stages of infection, thereby triggering the signaling pathway required for ROS accumulation. ROS species accumulate during infection, resulting in hypersensitive response and cell wall reinforcement (Zhu et al., 2020). In plants, pathogen infection activates a series of defense responses, including the synthesis of PR proteins. β-1,3-glucanase is a type of PR protein that defends against fungal infection by hydrolyzing β-1,3-glucan, a fungal cell wall polymer, to inhibit fungal growth (Fujimori et al., 2016). Two DEGs encoding β-1,3-glucanase-related genes were identified in our study; one DEG (TraesCS3A02G483000) was significantly upregulated in resistant cultivar (Gannong 2) and downregulated in susceptible cultivar (Shida 1), and the other one followed the opposite expression pattern. Several studies have shown that the chitinase activity of resistant varieties was higher than that of susceptible varieties at the early stage of pathogen infection (Zhao, 2004). In our study, DEGs related to chitinase were all downregulated in resistant cultivar Gannong 2 during *Pst* infection. We hypothesize that these chitinase-related DEGs may also have been expressed at a very early stage (germinating spores) in the resistant triticale cultivar. Previous studies have indicated that deregulation of CaM/CML genes or loss of CaM/CML function strongly affects immune responses (Cheval et al., 2013). In our study, three CaM/CMLs were markedly downregulated in resistant cultivar (Gannong 2) after *Pst* infection, whereas the other two were markedly upregulated. PR1 proteins are considered to be markers for an enhanced defensive state conferred by pathogen-induced SAR (Sekhon et al., 2006). Three DEGs were annotated as PR1 proteins in our study. The above DEGs likely participated in the defense response of the susceptible cultivar and the resistance response of the resistant cultivar, which suggests that triticale synthetically use multiple synthetic pathways to prevent fungal infection.

When plants are invaded by pathogens, some TFs can bind to specific *cis*-regulatory elements present in target gene promoters and activate downstream transduction pathways (Singh et al., 2002). TFs can therefore induce many downstream genes in plants to improve their resistance to disease (Tsuda and Somssich, 2015). TF families implicated in the regulation of plant defense mechanisms against pathogens mainly include WRKY, MYB, AP2/ERF, and bZIP (Alves et al., 2014). In the present study, members of NAC, WRKY, FAR1, AP2/ERF-ERF, GRAS, and MADS-MIKC TF families were differentially expressed in response to *Pst* infection. The largest number of differentially expressed TFs was NAC family members. Although the primary role of the NAC family is regulating responses to abiotic stresses such as drought and salinity, several NAC TFs in Arabidopsis (*At*ANAC019, *At*ANAC055 and *At*ANAC072) have also been identified as important immune components (Tsuda and Somssich, 2015). *Ta*NAC1 acts as a negative regulator of yellow rust resistance in wheat and is able to regulate JA and SA signal defense pathways (Wang et al., 2015). In our study, most of the NACs in the resistant cultivar (Gannong 2) were downregulated compared with the susceptible cultivar (Shida 1). The above-mentioned study demonstrated the importance of NAC TFs in plant disease resistance, a conclusion also confirmed by our observation of a large number of downregulated NAC genes in the resistant triticale cultivar after infection with *Pst*. WRKY is the most reported transcription factor associated with the disease resistance of plants, which plays an important regulatory role in the induction of defense responses to pathogens, and the major function of numerous distinct WRKY members in host immunity has been firmly established in Arabidopsis, barley, and rice (Pandey and Somssich, 2009). In addition, Cheng et al. (2015) have reported that the transcriptional regulation of *WRKY45*, *WRKY13*, and *WRKY42* is involved in the blast resistance of rice. In the present study, we identified eight WRKYs, six and seven of which were upregulated at 10 and 20 days after inoculation, respectively. Rice *OsWRKY45* has been reported to play a pivotal role in fungal disease resistance by mediating SA signaling (Shimono et al., 2012). In regards to the differentially expressed TFs in our study, some of these TFs may regulate the yellow rust resistance of triticale, which is consistent with the previous reports of positive and negative responses of TFs of wheat, rice, and other plants to fungal disease. The relationships of these TFs in signaling pathways of triticale against *Pst* are unknown, however, and their functions in triticale yellow rust resistance require further study.

The growth, development, and disease resistance-related functions of receptor-like protein kinases (RLKs), which are commonly found in plants, are a focus of increasing attention (Sun et al., 2004). As signaling molecule receptors, RLKs play an important role in signal transduction. Most of these kinases exist at the nodes of various pathways, thereby forming a complex network with their upstream and downstream proteins (Li et al., 2008). The accumulation of intracellular signaling molecules induces specific phosphorylation and dephosphorylation cascades, with the ensuing metabolic changes leading to specific plant responses that include the activation of cadres of genes involved in particular defense responses (Gachomo et al., 2003). Some resistance genes are homologous to RLK genes in plants. Most RLKs involved in plant resistance belong to the LRR-RLK sub-family; examples include rice *Xa21* involved in bacterial pathogen resistance (Song et al., 1995) and Arabidopsis *FLS2* related to flagellin perception (Robatzek et al., 2006), which plays an important role in plant resistance response and pathogen recognition. Most RLKs in plants are serine/threonine kinases (Li et al., 2008). We also identified many serine/threonine-protein kinases in this study, most of which were found to be significantly upregulated in the resistant cultivar (Gannong 2) and downregulated in the susceptible cultivar (Shida 1), which may be why Gannong 2 can inhibit pathogen infection and exhibit high disease resistance. In addition, five kinase genes had significantly different expressions before (10 days) and after (20 days) sporulation, thus showing that they may have some functions in disease defense at a later stage. Furthermore, pathogens use effectors to interfere with the plant-based PTI defense system; on the other hand, plants have developed ETI responses based on effector recognition (Boller and Felix, 2009). Several disease-resistance proteins, such as RPM1, RPP13, and RGAs, were upregulated in resistant cultivar (Gannong 2) and may be involved in ETI. Plants maintain many resistance proteins (*R* genes) that directly or indirectly recognize effectors released from pathogens and initiate ETI (Kim et al., 2012).

Overall, this study represents the first step toward understanding of the molecular mechanisms involved in resistance to *Pst* in triticale. Our results provide a molecular basis for the elucidation of triticale–*Pst* interactions and can serve as a candidate gene resource for yellow rust resistance breeding in triticale.

## Data Availability Statement

The datasets presented in this study can be found in online repositories. The names of the repository/repositories and accession number(s) can be found at: NCBI—SRR18675448, SRR18675447, SRR18675446, SRR18675445, SRR18675444, SRR18675443, SRR18675442, SRR18675441.

## Author Contributions

FZ conducted the experiments, analyzed the data, and wrote the manuscript. KN participated in qRT-PCR experiment, provided technical support, and revised the manuscript critically. XT and WD provided the experimental materials. WD conceived the experiment and manuscript revision. All authors contributed to the article and approved the submitted version.

## Funding

This study was financially supported by Science and Technology Program of Gansu Province (20YF8NA129 and 21ZD4NA012), Tibet Forage Industry Project (XZ202101ZD0003N), and the National Natural Science Foundation of China (31760702).

## Conflict of Interest

The authors declare that the research was conducted in the absence of any commercial or financial relationships that could be construed as a potential conflict of interest.

## Publisher’s Note

All claims expressed in this article are solely those of the authors and do not necessarily represent those of their affiliated organizations, or those of the publisher, the editors and the reviewers. Any product that may be evaluated in this article, or claim that may be made by its manufacturer, is not guaranteed or endorsed by the publisher.

## Footnotes

^1^ http://urgi.versailles.inra.fr/download/iwgsc/IWGSC_RefSeq_Annotations/v1.1/

^2^ http://www.biocloud.net//

## References

[ref1] Altschul S. F. Madden T. L. Schaffer A. A. Zhang J. H. Zhang Z. Miller W.. (1997). Gapped BLAST and PSI-BLAST: a new generation of protein database search programs. Nucleic Acids Res. 25, 3389–3402. doi: 10.1093/nar/25.17.3389, PMID: 9254694 PMC146917

[ref2] Alves M. S. Dadalto S. P. Gonçalves A. B. de Souza G. B. Barros V. A. Fietto L. G. (2014). Transcription factor functional protein-protein interactions in plant defense responses. Proteomes 2, 85–106. doi: 10.3390/proteomes2010085, PMID: 28250372 PMC5302731

[ref3] Audenaert K. Troch V. Landschoot S. Haesaert G. (2014). Biotic stresses in the anthropogenic hybrid triticale (×*Triticosecale* Wittmack): current knowledge and breeding challenges. Eur. J. Plant Pathol. 140, 615–630. doi: 10.1007/s10658-014-0498-2

[ref4] Bari R. Jones J. D. G. (2009). Role of plant hormones in plant defence responses. Plant Mol. Biol. 69, 473–488. doi: 10.1007/s11103-008-9435-0 19083153

[ref5] Baron V. S. Juskiw P. E. Aljarrah M. (2015). “Triticale as a Forage,” in Triticale. ed. Eudes F. (Canada: Springer, Cham Press), 189–212.

[ref6] Boller T. Felix G. (2009). A renaissance of elicitors: perception of microbe-associated molecular patterns and danger signals by pattern-recognition receptors. Annu. Rev. Plant Biol. 60, 379–406. doi: 10.1146/annurev.arplant.57.032905.105346, PMID: 19400727

[ref7] Bozkurt O. Unver T. Akkaya M. S. (2007). Genes associated with resistance to wheat yellow rust disease identified by differential display analysis. Physiol Mol Plant P. 71, 251–259. doi: 10.1016/j.pmpp.2008.03.002

[ref8] Camehl I. Sherameti I. Seebald E. Johnson J. M. Oelmüller R. (2013). “Role of defense compounds in the beneficial interaction between *Arabidopsis thaliana* and *Piriformospora indica*,” in Piriformospora indica. eds. Varma A. Kost G. Oelmüller R. (Springer, Berlin, Heidelberg Press), 239–250.

[ref9] Chen W. Q. Wellings C. Chen X. M. Kang Z. S. Liu T. G. (2014). Wheat stripe (yellow) rust caused by *Puccinia striiformis* f. sp. tritici. Mol. Plant Pathol. 15, 433–446. doi: 10.1111/mpp.12116, PMID: 24373199 PMC6638732

[ref10] Cheng H. T. Liu H. B. Deng Y. Xiao J. H. Li X. H. Wang S. P. (2015). The *WRKY45-2 WRKY13 WRKY42* transcriptional regulatory cascade is required for rice resistance to fungal pathogen. Plant Physiol. 167, 1087–1099. doi: 10.1104/pp.114.256016, PMID: 25624395 PMC4348788

[ref11] Cheon J. Fujioka S. Dilkes B. P. Choe S. (2013). Brassinosteroids regulate plant growth through distinct signaling pathways in Selaginella and Arabidopsis. PLoS One 8:e81938. doi: 10.1371/journal.pone.0081938, PMID: 24349155 PMC3862569

[ref12] Cheval C. Aldon D. Galaud J. P. Ranty B. (2013). Calcium/calmodulin-mediated regulation of plant immunity. Biochim. Biophys. Acta 1833, 1766–1771. doi: 10.1016/j.bbamcr.2013.01.031, PMID: 23380707

[ref13] De Vleesschauwer D. Van Buyten E. Satoh K. Balidion J. Mauleon R. Choi I. R.. (2012). Brassinosteroids antagonize gibberellin- and salicylate-mediated root immunity in rice. Plant Physiol. 158, 1833–1846. doi: 10.2307/41496323, PMID: 22353574 PMC3320189

[ref14] Dobon A. Bunting D. C. Cabrera-Quio L. E. Uauy C. Saunders D. G. (2016). The host-pathogen interaction between wheat and yellow rust induces temporally coordinated waves of gene expression. BMC Genomics 17:380. doi: 10.1186/s12864-016-2684-4, PMID: 27207100 PMC4875698

[ref15] Fujimori N. Enoki S. Suzuki A. Naznin H. A. Shimizu M. Suzuki S. (2016). Grape apoplasmic β-1,3-glucanase confers fungal disease resistance in Arabidopsis. Sci. Hortic. 200, 105–110. doi: 10.1016/j.scienta.2016.01.008

[ref16] Gachomo E. W. Shonukan O. O. Kotchoni S. O. (2003). The molecular initiation and subsequent acquisition of disease resistance in plants. Afr. J. Biotechnol. 2, 26–32. doi: 10.5897/AJB2003.000-1005

[ref17] Glazebrook J. (2005). Contrasting mechanisms of defense against biotrophic and necrotrophic pathogens. Annu. Rev. Phytopathol. 43, 205–227. doi: 10.1146/annurev.phyto.43.040204.135923, PMID: 16078883

[ref18] González J. M. Muñiz L. M. Jouve N. (2005). Mapping of QTLs for androgenetic response based on a molecular genetic map of × *Triticosecale* Wittmack. Genome 48, 999–1009. doi: 10.1139/g05-064, PMID: 16391669

[ref19] Gyawali S. Verma R. P. S. Kumar S. Bhardwaj S. C. Gangwar O. P. Selvakumar R.. (2017). Seedling and adult-plant stage resistance of a world collection of barley genotypes to stripe rust. J. Phytopathol. 166, 18–27. doi: 10.1111/jph.12655

[ref20] Hao Y. B. Wang T. Wang K. Wang X. J. Fu Y. P. Huang L. L.. (2016). Transcriptome analysis provides insights into the mechanisms underlying wheat plant resistance to stripe rust at the adult plant stage. PLoS One 11:e0150717. doi: 10.1371/journal.pone.0150717, PMID: 26991894 PMC4798760

[ref21] He Y. Zhang H. Sun Z. Li J. Hong G. Zhu Q.. (2017). Jasmonic acid-mediated defense suppresses brassinosteroid-mediated susceptibility to Rice black streaked dwarf virus infection in rice. New Phytol. 214, 388–399. doi: 10.1111/nph.14376 27976810

[ref22] Huang F. Li W. Z. Chen T. Q. Xiong S. J. Wang W. Zhang L. Y. (2014). Identification of the resistance to wheat stripe rust of Guizhou major wheat cultivars (lines). Guizhou Agric Sci. 42, 81–84+88. doi: 10.3969/j.issn.1001-3601.2014.02.021

[ref23] Huang L. Liu T. G. Liu B. Gao L. Luo P. G. Chen W. Q. (2019). Resistance evaluation of 197 Chinese wheat core germplasms to a new stripe rust race, CYR34. Plant Prot. 45, 148–154. doi: 10.16688/j.zwbh.2018067

[ref24] Jones J. D. G. Dangl J. L. (2006). The plant immune system. Nature 444, 323–329. doi: 10.1038/nature05286 17108957

[ref25] Jones D. A. Takemoto D. (2004). Plant innate immunity—direct and indirect recognition of general and specific pathogen-associated molecules. Curr. Opin. Immunol. 16, 48–62. doi: 10.1016/j.coi.2003.11.016, PMID: 14734110

[ref26] Kim D. Langmead B. Salzberg S. L. (2015). HISAT: a fast spliced aligner with low memory requirements. Nat. Methods 12, 357–360. doi: 10.1038/nmeth.3317, PMID: 25751142 PMC4655817

[ref27] Kim J. Lim C. J. Lee B. W. Choi J. P. Oh S. K. Ahmad R.. (2012). A genome-wide comparison of NB-LRR type of resistance gene analogs (RGA) in the plant kingdom. Mol. Cells 33, 385–392. doi: 10.1007/s10059-012-0003-8, PMID: 22453776 PMC3887800

[ref28] Kinkema M. Fan W. H. Dong X. N. (2000). Nuclear localization of NPR1 is required for activation of PR gene expression. Plant Cell 12, 2339–2350. doi: 10.2307/3871233, PMID: 11148282 PMC102222

[ref29] Li L. Y. Jin M. J. Liu Q. Liu G. Z. (2008). Function and substrate identification of plant receptor-like kinases. Chin. J. Biochem. Mol. Biol. 24, 113–119. doi: 10.3969/j.issn.1007-7626.2008.02.006

[ref30] Liu J. L. Liu X. L. Dai L. Y. Wang G. L. (2007). Recent progress in elucidating the structure, function and evolution of disease resistance genes in plants. J. Genet. Genomics 34, 765–776. doi: 10.1016/S1673-8527(07)60087-3, PMID: 17884686

[ref31] Livak K. J. Schmittgen T. D. (2001). Analysis of relative gene expression data using real time quantitative PCR and the 2^−ΔΔCT^ method. Methods 25, 402–408. doi: 10.1006/meth.2001.1262 11846609

[ref32] Losert D. Maurer H. P. Leiser W. L. Würschum T. (2017). Defeating the warrior: genetic architecture of triticale resistance against a novel aggressive yellow rust race. Theor. Appl. Genet. 130, 685–696. doi: 10.1007/s00122-016-2843-7, PMID: 28039516

[ref33] Mao X. Z. Cai T. Olyarchuk J. G. Wei L. P. (2005). Automated genome annotation and pathway identification using the KEGG Orthology (KO) as a controlled vocabulary. Bioinformatics 21, 3787–3793. doi: 10.2307/1592215, PMID: 15817693

[ref34] Pandey S. P. Somssich I. E. (2009). The role of WRKY transcription factors in plant immunity. Plant Physiol. 150, 1648–1655. doi: 10.1104/pp.109.138990, PMID: 19420325 PMC2719123

[ref35] Peng P. Zhao J. Zhu Y. Y. Asami T. Li J. M. (2010). A direct docking mechanism for a plant GSK3-like kinase to phosphorylate its substrates. J. Biol. Chem. 285, 24646–24653. doi: 10.1074/jbc.M110.142547, PMID: 20522560 PMC2915701

[ref36] Pertea M. Pertea G. M. Antonescu C. M. Chang T. C. Mendell J. T. Salzberg S. L. (2015). StringTie enables improved reconstruction of a transcriptome from RNA-seq reads. Nat. Biotechnol. 33, 290–295. doi: 10.1038/nbt.3122, PMID: 25690850 PMC4643835

[ref37] Pieterse C. M. J. Van der Does D. Zamioudis C. Leon-Reyes A. Van Wees S. C. M. (2012). Hormonal modulation of plant immunity. Annu. Rev. Cell Dev. Biol. 28, 489–521. doi: 10.1146/annurev-cellbio-092910-154055, PMID: 22559264

[ref38] Ramamoorthy R. Jiang S. Y. Kumar N. Venkatesh P. N. Ramachandran S. (2008). A comprehensive transcriptional profiling of the WRKY gene family in rice under various abiotic and phytohormone treatments. Plant Cell Physiol. 49, 865–879. doi: 10.1093/pcp/pcn061, PMID: 18413358

[ref39] Ren Y. Li S. R. Xia X. C. Zhou Q. He Y. J. Wei Y. M.. (2015). Molecular mapping of a recessive stripe rust resistance gene *yrMY37* in Chinese wheat cultivar Mianmai 37. Mol. Breeding. 35, 97. doi: 10.1016/S2095-3119(14)60781-4

[ref40] Robatzek S. Chinchilla D. Boller T. (2006). Ligand-inducedendocytosis of the pattern recognition receptor *FLS2* in Arabidopsis. Genes Dev. 20, 537–542. doi: 10.1101/gad.366506, PMID: 16510871 PMC1410809

[ref41] Robert-Seilaniantz A. Grant M. Jones J. D. G. (2011). Hormone crosstalk in plant disease and defense: more than just jasmonate-salicylate antagonism. Annu. Rev. Phytopathol. 49, 317–343. doi: 10.1146/annurev-phyto-073009-114447, PMID: 21663438

[ref42] Roelfs A. P. Singh R. P. Saari E. E. (1992). Rust Disease of Wheat: Concepts and Methods of Disease Management. Mexico: CIMMYT Press.

[ref43] Sekhon R. S. Kuldau G. Mansfield M. Chopra S. (2006). Characterization of Fusarium-induced expression of flavonoids and PR genes in maize. Physiol. Mol. Plant P. 69, 109–117. doi: 10.1016/j.pmpp.2007.02.004

[ref44] Shimono M. Koga H. Akagi A. Hayashi N. Goto S. Sawada M.. (2012). Rice *WRKY45* plays important roles in fungal and bacterial disease resistance. Mol. Plant Pathol. 13, 83–94. doi: 10.1111/j.1364-3703.2011.00732.x, PMID: 21726399 PMC6638719

[ref45] Singh K. B. Foley R. C. Oate-Sánchez L. (2002). Transcription factors in plant defense and stress responses. Curr. Opin. Plant Biol. 5, 430–436. doi: 10.1016/S1369-5266(02)00289-3 12183182

[ref46] Sodkiewicz W. Strzembicka A. Sodkiewicz T. Majewska M. (2009). Response to stripe rust (*Puccinia striiformis, Westend*. f. Sp. *tritici*) and its coincidence with leaf rust resistance in hexaploid introgressive triticale lines with Triticum monococcum genes. J. Appl. Genet. 50, 205–211. doi: 10.1007/BF03195674, PMID: 19638675

[ref47] Song W. Y. Wang G. L. Chen L. L. Kim H. S. Pi L. Y. Hoisten T.. (1995). A receptor kinase-like protein encoded by the rice disease resistance gene, *Xa21*. Science 270, 1804–1806. doi: 10.1126/science.270.5243.1804, PMID: 8525370

[ref48] Spoel S. H. Dong X. N. (2008). Making sense of hormone crosstalk during plant immune responses. Cell Host Microbe 3, 348–351. doi: 10.1016/j.chom.2008.05.009, PMID: 18541211

[ref49] Sun X. H. Lu T. G. Jia S. R. Huang D. F. (2004). Genome-wide bioinformatics analysis of the rice receptor-like kinase family. Sci. Agric. Sin. 37, 322–327. doi: 10.3321/j.issn:0578-1752.2004.03.002

[ref50] Trapnell C. Williams B. A. Pertea G. Mortazavi A. Kwan G. van Baren M. J.. (2010). Transcript assembly and quantification by RNA-Seq reveals unannotated transcripts and isoform switching during cell differentiation. Nat. Biotechnol. 28, 511–515. doi: 10.1038/nbt.1621, PMID: 20436464 PMC3146043

[ref51] Tsuda K. Somssich I. E. (2015). Transcriptional networks in plant immunity. New Phytol. 206, 932–947. doi: 10.1111/nph.13286 25623163

[ref52] Wang F. T. Lin R. M. Feng J. Chen W. Q. Qiu D. W. Xu S. C. (2015). TaNAC1 acts as a negative regulator of stripe rust resistance in wheat, enhances susceptibility to *Pseudomonas syringae*, and promotes lateral root development in transgenic *Arabidopsis thaliana*. Front. Plant Sci. 6:108. doi: 10.3389/fpls.2015.00108, PMID: 25774162 PMC4342887

[ref53] Xue J. Bao Y. Y. Li B. L. Cheng Y. B. Peng Z. Y. Liu H.. (2010). Transcriptome analysis of the brown planthopper *Nilaparvata lugens*. PLoS One 5:e14233. doi: 10.1371/journal.pone.0014233, PMID: 21151909 PMC2997790

[ref54] Yang C. Li L. Y. Feng A. Q. Zhu X. Y. Li J. X. (2014). Transcriptional profiling of the responses to infection by the false smut fungus *Ustilaginoidea virens* in resistant and susceptible rice varieties. Can. J. Plant Pathol. 36, 377–388. doi: 10.1080/07060661.2014.927925

[ref55] Young M. D. Wakefield M. J. Smyth G. K. Oshlack A. (2010). Gene ontology analysis for RNA-seq: accounting for selection bias. Genome Biol. 11:R14. doi: 10.1186/gb-2010-11-2-r14, PMID: 20132535 PMC2872874

[ref56] Yuan G. S. He X. J. Li H. Xiang K. Liu L. Zou C. Y.. (2019). Transcriptomic responses in resistant and susceptible maize infected with *Fusarium graminearum*. Crop J. 8, 153–163. doi: 10.1016/j.cj.2019.05.008

[ref57] Zhang H. Yang Y. Z. Wang C. Y. Liu M. Li H. Fu Y.. (2014). Large-scale transcriptome comparison reveals distinct gene activations in wheat responding to stripe rust and powdery mildew. BMC Genomics 15:898. doi: 10.1186/1471-2164-15-898, PMID: 25318379 PMC4201691

[ref58] Zhao H. (2004). Chitinase and plant protection. J. Hebei Agric. Sci. 8, 78–84. doi: 10.3969/j.issn.1088-1631.2004.02.019

[ref59] Zhu X. B. Ze M. Chern M. Chen X. W. Wang J. (2020). Deciphering rice lesion mimic mutants to understand molecular network governing plant immunity and growth. Rice Sci. 27, 278–288. doi: 10.1016/j.rsci.2020.05.004

